# Bacterial communities of fungus-growing ant queens are species-specific and suggest vertical transmission

**DOI:** 10.1371/journal.pone.0306011

**Published:** 2025-10-07

**Authors:** Victoria A. Sadowski, Panagiotis Sapountzis, Pepijn W. Kooij, Jacobus J. Boomsma, Rachelle M. M. Adams

**Affiliations:** 1 Department of Evolution, Ecology, and Organismal Ecology, The Ohio State University, Columbus, Ohio, United State of America; 2 Centre for Social Evolution, Department of Biology, University of Copenhagen, Copenhagen, Denmark; 3 UMR 0454 MEDIS, INRAE- UCA, Clermont-Ferrand, France; 4 Department of General and Applied Biology, São Paulo State University (UNESP), Rio Claro, Brazil; 5 Smithsonian Tropical Research Institute, Ancon, Panamá, República de Panamá; Universidade de São paulo, BRAZIL

## Abstract

Multipartite symbioses are inherently complex, involving dynamic ecological interactions between organisms with intertwined yet distinct evolutionary histories. The fungus-growing (attine) ants facilitate maintenance of a symbiotic species network through maternal vertical transmission of a fungal symbiont. While the gut microbiomes of fungus-growing ant species are remarkably simple, their fungus gardens support diverse microbial communities. Here, we focus on the garden pellet stored in the nest-founding queen’s infrabuccal pocket—a food filter in the head that allows ants to expel large particles. The pellet is an inoculate of the new fungal garden but also contains other microbes. We used 16S rRNA gene amplicon sequencing to reconstruct the extent of vertical transmission of bacteria to new gardens via queen pellets in four sympatric fungus-growing ant species from Central Panama (*Atta sexdens*, *Atta cephalotes*, *Acromyrmex echinatior,* and *Mycetomoellerius mikromelanos*). We also characterized the bacterial communities associated with queen eggs and tissues (mesosomas, guts and ovaries) to assess whether queens are likely to transmit symbiotic bacteria, such as cuticular Actinomycetota and endosymbionts (*Wolbachia*, *Mesoplasma*, and *Spiroplasma*). We made within and between species comparisons, focusing on three hypotheses: (H1) Queens vertically transmit garden-associated bacteria in the garden pellet. (H2) Fungus-growing ant-associated bacteria are maintained through vertical transmission by queens. (H3) Vertically transmitted bacterial communities have host ant species-specificity. While we found mixed evidence for vertical transmission of garden bacteria, our results support maternal transmission as an important route for ant-associated symbionts. The ant species-specificity we see in queen bacterial microbiota mirrors patterns of known symbiont presence in workers from previous studies. Overall, our results suggest that vertical transmission of bacterial associates is mediated by the ant hosts, however the mechanism behind bacterial acquisition before a mating flight and dispersal is not yet understood.

## Introduction

A fundamental question in the study of symbioses is how mutualistic partners associate in manners that allow co-adaptation [1–3]. Theory predicts that faithful vertical transmission allows positive selective feedback [4,5], yet there are also many examples of horizontal transmission facilitating reliable partner association [3,6–8]. The fungus-growing attine ants (Hymenoptera: Formicidae: Attini: Attina) maintain a nutritional mutualism with mainly *Leucoagaricus* and *Leucocoprinus* fungal cultivars (Basidiomycota: Agaricomycetes: Agaricales: Agaricaceae) reared in underground gardens and provisioned with fresh plant material, insect frass, and/or partly degraded plant matter [9–11]. Leafcutters in the genera [12–17] *Atta* and *Acromyrmex* are the most well-known fungus-growing ants, having a considerable ecological impact with thousands to millions of workers often traveling over 100 meters to collect fresh plant material to feed their large fungal gardens [9,18]. Sympatric *Atta* and *Acromyrmex* species have different preferences for woody vs. herbaceous plants, respectively [19] and avoid competition by occupying different niches in the same general area. For example, *Atta cephalotes* forages on plants in the forest while *Atta sexdens* prefers more open sunlit areas [19]. In contrast to leafcutters, most fungus-growing ant species are less conspicuous and not as reliant on fresh plant material. These species travel shorter distances and collect a wider variety of forage such as insect frass and partly degraded organic matter for their smaller fungal gardens [10]. Irrespective of these ecological distinctions, all fungus-growing ants are obligately dependent on their gardens which function as an external digestive system that degrades the organic material the ants provide. With a diverse assemblage of microbes within the garden matrix, the carbon-rich forage material is processed to provide a variety of accessible nutrients for the ants [12–16].

Gynes—unmated female reproductives destined to become queens—provide a vertical transmission route for microbes in this system. Before their mating flight, they pluck a garden fragment and store it as a pellet in the infrabuccal pocket, an oral cavity that allows ants to filter and expel solid food particles [20]. Newly mated queens use this infrabuccal pellet (referred to as pellet hereafter) as garden inoculum to transfer fungal cultivar and associated microbes from the natal nest to a new incipient colony [9]. The gardens of *Atta* and *Acromyrmex* leafcutter ant species are known to host similar bacterial taxa [21], suggesting that there is selection for certain microbes in the fungal gardens. However, direct evidence of vertical transmission of garden bacteria is lacking and horizontal acquisition from the environment cannot be excluded. How unmated gynes choose their pellet is unknown, though they are likely under positive selective pressure to transmit beneficial garden bacteria and to avoid transmission of pathogens (but see [6,22]). Gynes might preferentially choose a pellet inoculum with a high abundance of bacterial symbionts. Alternatively, they may randomly acquire maternal fungus garden. Pellet surveys so far have suggested that both the fungal cultivar and the associated bacteria may indeed be vertically transmitted (i.e., parent to offspring). For example, the infrabuccal pellets in newly mated queens of the leafcutter genus *Atta* contain bacteria that are closely related to bacteria commonly found in mature gardens [15,23,24]. Although, no studies have sampled pellets from *Acromyrmex* or *Mycetomoellerius* and garden-associated bacterial communities are not well characterized.

Characterization of fungus-growing ant worker microbiomes has mostly focused on cuticular Actinomycetota which grow on worker mesosomas (i.e., alitrunk, truncus, or thorax) and produce antimicrobial compounds effective against *Escovopsis* fungal parasites and other pathogens [25–29]. Additionally, the presence of visible cuticular Actinomycetota in fungus-growing ant species correlates with gut microbiota composition [30]. In the phylogenetic crown group, also referred to as the higher attine ants, worker gut bacterial communities have very low diversity compared to fungal gardens, similar to other insects with specialized diets [31,32], and are usually dominated by a few OTUs belonging to *Wolbachia* (Pseudomonadota: Alphaproteobacteria: Rickettsiales: Ehrlichiaceae) and the bacterial orders Rhizobiales and Mycoplasmatales [30]. These abundant bacteria persist when ant colonies are transferred from the field to the lab, suggesting they are functionally important symbionts [30]. *Wolbachia*’s role remains elusive although these bacteria are consistently found in somatic tissues of *Acromyrmex* species [30,33,34]. *Mesoplasma* (Bacillota: Mollicutes: Mycoplasmatales: Mycoplasmataceae; also known as *Edwardiiplasma* and *Entomoplasma* [35,36]) is primarily associated with leafcutter ant species while non-leafcutter attines also associate with *Spiroplasma* (Bacillota: Mollicutes: Mycoplasmatales: Spiroplasmataceae) [30]. These Mycoplasmatales symbionts likely provide metabolic services to their attine host ants by converting citrate into easily accessible acetate and by recycling excess—fungal cultivar produced—arginine into ammonia [37,38]. Aside from these endosymbionts, Rhizobiales can form biofilms in the guts of leafcutter ants, possibly contributing to mutualism-stability by nutrient exchange with other gut symbionts [39,40]. However, it remains unclear whether these bacterial symbionts are vertically transmitted by queens. Previous work has shown that in *Acromyrmex*, *Wolbachia* is vertically transmitted via eggs, but Mycoplasmatales (*Mesoplasma* and *Spiroplasma*) symbionts were instead primarily transmitted from older to newly emerged workers [41]. Mycoplasmatales have also been detected in the eggs of the non-leafcutter attine *Sericomyrmex amabilis* and in the unmated gynes of its social parasite [42], indicating the potential for a mixed mode of transmission; vertically from parent to offspring and horizontally between ant species. While recent studies have begun to establish the functionality of these symbioses in attine ant workers, further investigation is needed to better understand their transmission dynamics.

In this study we used 16S ribosomal RNA gene metabarcoding to characterize bacterial microbiota in four fungus-growing ant species from the Panama Canal region: *Atta sexdens* (Linnaeus, 1758) [43], *Atta cephalotes* (Linnaeus, 1758) [43], *Acromyrmex echinatior* (Forel, 1899) [44] and *Mycetomoellerius mikromelanos* [45]. We sampled unmated gyne tissue (used as a proxy for mated queens), pellets, and garden from mature colonies in all four species. We also sampled tissue from young newly mated queens, garden and eggs in *A. sexdens* and *M. mikromelanos* colonies. In our hypotheses we use “queens” to refer to unmated gynes and mated queens. In the following sections, we specify either gyne or queen samples where appropriate.

First, we made within-species comparisons to test the hypothesis (H1) that attine queens vertically transmit garden-associated bacteria through their pellet inocula. Under this hypothesis we expected that fungal pellet bacterial communities would consistently have the same bacterial taxa found in fungus gardens. We also hypothesized (H2) that ant-associated bacteria are vertically transmitted by the queens themselves. Under this hypothesis we expected that known gut and endosymbionts (*Wolbachia*, *Mesoplasma* and *Spiroplasma*) and cuticular Actinomycetota (in *Acromyrmex* and *Mycetomoellerius*) would be found in tissues of dispersing queens as well as in eggs of mated queens. We further used a comparative framework to explore bacterial community diversity between the four sympatric ant species. Here, we hypothesized (H3) that vertically transmitted microbiomes should have signatures reflecting host species-specificity. Under this hypothesis, we expected that queens of (non-leafcutter) *M. mikromelanos* would host unique bacteria relative to the three more closely related leafcutter ant species [11].

## Results

### Alpha-diversity and community composition within species

Analysis of rarefaction curves at 97% sequence similarity indicated that sequencing coverage was sufficient, apart from a small number of samples with exceptionally high diversity (S1 Fig). Alpha-diversity, or within-sample species richness and diversity, was assessed using the Inverse Simpson index, which showed that diversity was variable across sample types within ant species (S2 Fig and S5 Table). There was no significant difference in alpha-diversity in comparing ovaries to eggs, nor pellets to garden within each species (S2 Fig and S5 Table). The composition of bacterial phyla was variable across sample types and among ant species (S3 Fig). All bacterial communities were composed mostly of Pseudomonadota (Proteobacteria), Bacillota (Firmicutes), Actinomycetota (Actinobacteria) and Bacteroidota (Bacteroidetes) (S3 Fig). Pseudomonadota and Bacillota were particularly dominant in the bacterial communities of *Acromyrmex echinatior* (S3 Fig), which was largely due to the high abundance of *Wolbachia* and Mycoplasmatales symbionts in this species.

### Vertical transmission of garden-derived bacteria

**Fig 1:**
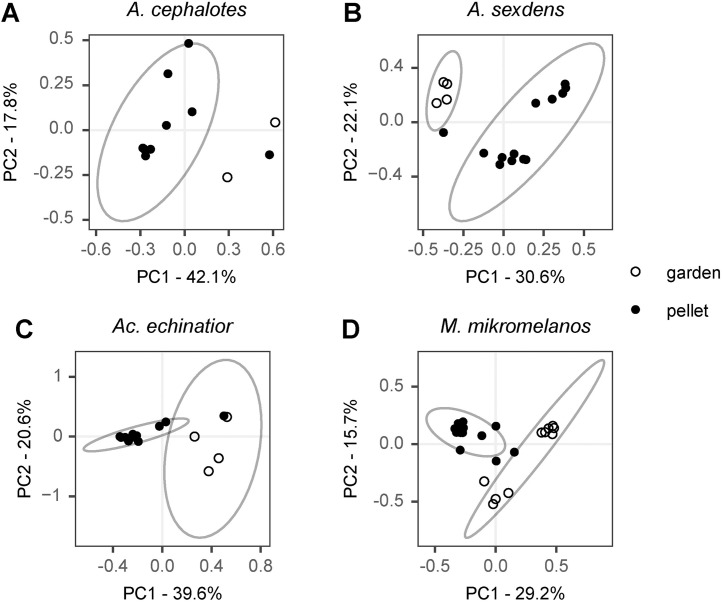
Within-species comparisons of pellet and garden beta-diversity using Weighted Unifrac Principal Coordinate Analysis (PCoA). (A) *Atta cephalotes,* (B) *Atta sexdens*, (C) *Acromyrmex echinatior* and (D) *Mycetomoellerius mikromelanos*. Ellipses represent 95% confidence intervals. The sample size for *A. cephalotes* garden samples was too low to calculate a statistical ellipse, but pellets and garden microbiota were different in all four ant species (p < 0.05).

**Fig 2:**
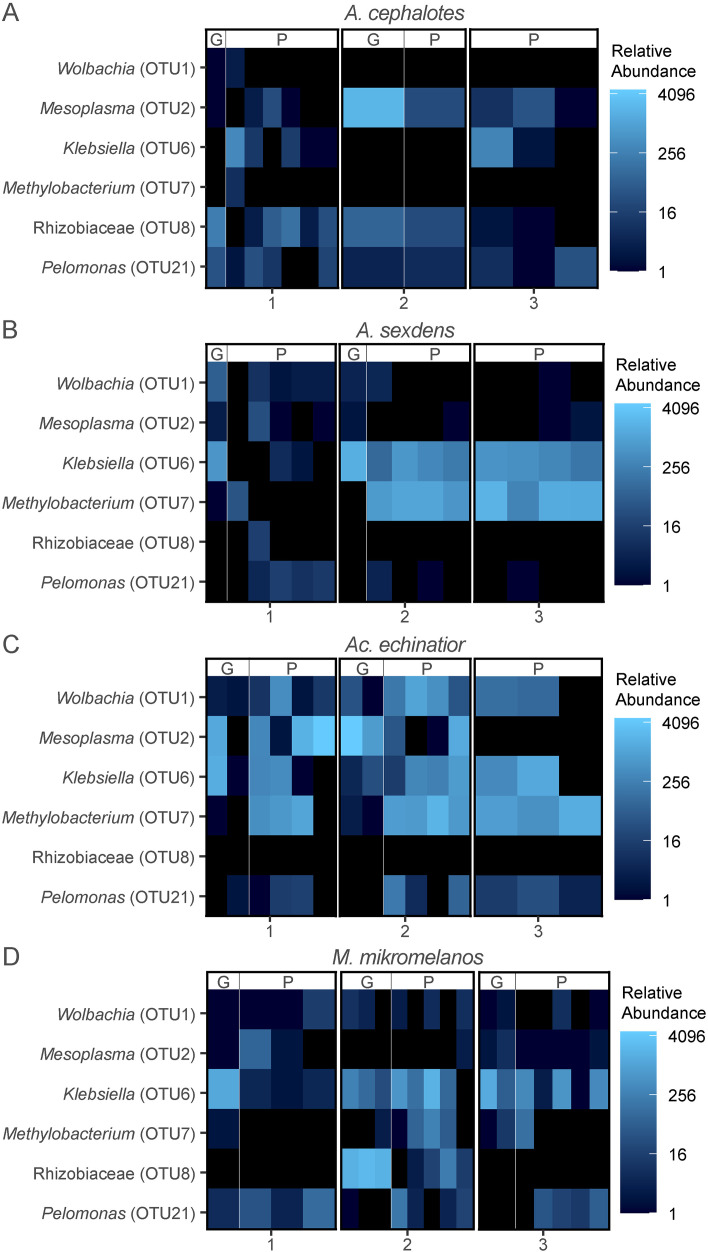
Heatmap depicting variation in abundance of bacterial OTUs common to both pellet and garden core microbiota in at least one ant species. (A) *Atta cephalotes,* (B) *Atta sexdens*, (C) *Acromyrmex echinatior* and (D) *Mycetomoellerius mikromelanos*. Garden and pellet samples are denoted with a “G” and “P” respectively. The color scale is based on log base 4 transformation of relative (proportional) abundance out of 5000 reads. See S1 Table for corresponding colony collection codes.

Using beta-diversity analyses to explore differences between sample types, we found that pellets and gardens hosted distinct bacterial communities in all four ant species (p < 0.05) (Fig 1, S6 and S7 Tables). Despite this, *Klebsiella* (OTU6) was a core taxon in both pellet and garden communities in *Ac. echinatior* and *M. mikromelanos* (S4 Table) and was present at a relatively high abundance in several of the pellet samples within all four ant species (Fig 2). Surprisingly, some known ant-associates (*Mesoplasma*, *Wolbachia* and Rhizobiales) were also consistently present and often relatively abundant in pellet and gardens for some ant species (Fig 2 and S4 Table). *Wolbachia* (OTU1) and *Methylobacterium* (OTU7) were core taxa in both pellet and garden communities in *Ac. echinatior* (S4 Table). In *Atta cephalotes*, *Mesoplasma* (OTU2), Rhizobiaceae (OTU8), and *Pelomonas* (OTU21) were shared core taxa in pellet and garden samples (S4 Table). However, *Atta sexdens* pellet and garden samples did not have core bacterial OTUs in common (S4 Table). The Rhizobiaceae OTU (OTU8) is closely related to bacteria from South American *Atta laevigata* guts (GenBank accession: KF250158.1) and to bacteria shared with other fungus-growing ants: *Sericomyrmex amabilis* and *Mycetomoellerius mikromelanos* (previously *zeteki*) and their social parasites (*Megalomyrmex symmetochus* and *Megalomyrmex adamsae* respectively) (GenBank accession: LC027777.1; Liberti et al. (2015)). Thus, our H1 hypothesis that newly mated queens vertically transmit garden bacteria was partially supported by the data we obtained, as only a select few OTUs are consistently present in both pellets and gardens within each ant species.

### Potential for vertical transmission of ant-derived bacteria

#### Transovarial transmission in mated queens.

**Fig 3:**
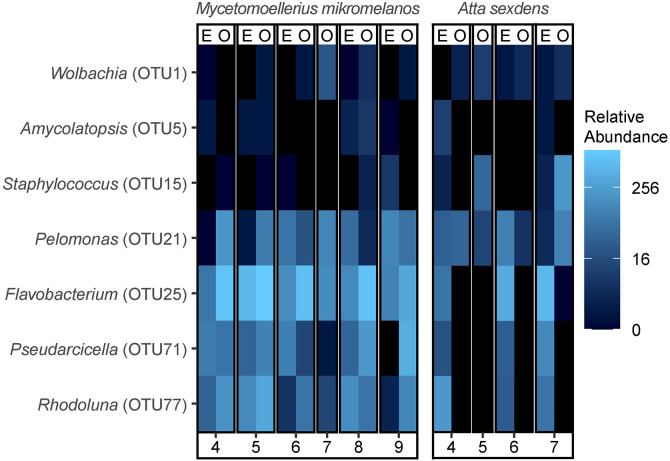
Heatmap depicting variation in abundance of bacterial OTUs common to both egg and ovary core microbiota in at least one ant species. Egg and ovary samples are denoted with an “E” and “O” respectively. The color scale is based on log base 4 transformation of relative (proportional) abundance out of 5000 reads. See S1 Table for corresponding colony collection codes.

For two ant species, we were able to sample young mated foundress queens and their eggs in addition to unmated gynes from older colonies. Despite the composition of egg and ovary bacterial communities being somewhat distinct (p < 0.05) (S4 Fig, S6 and S7 Tables), *Atta sexdens* and *M. mikromelanos* queens hosted some of the same bacterial taxa in their ovaries as were consistently found in their eggs, such as *Wolbachia* (OTU1) and *Amycolatopsis* (OTU5) (Fig 3 and S4 Table). However, neither *Spiroplasma*, *Mesoplasma* nor Rhizobiales were shared core taxa in the ovaries and eggs of either ant species (S4 Table).

#### Known bacterial symbionts present in queen and gyne tissue.

**Fig 4:**
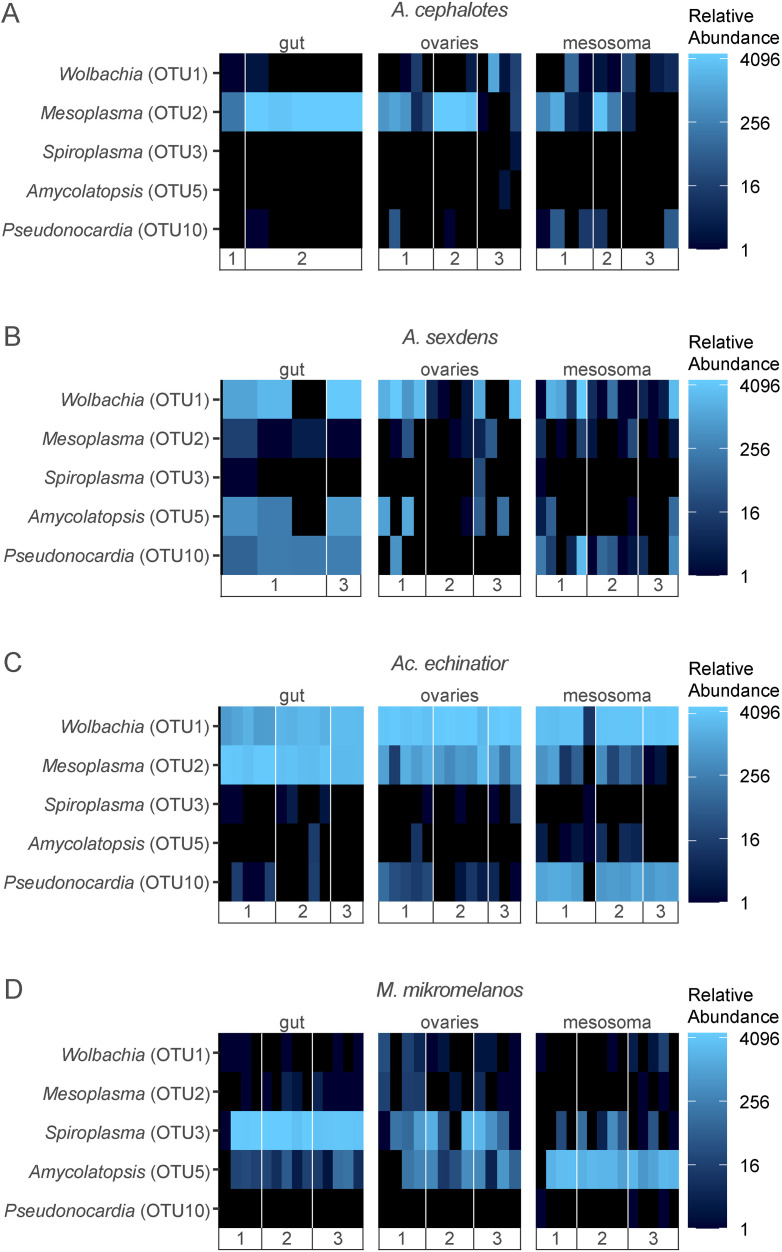
Heatmaps depicting variation in relative abundance of bacterial symbiont OTUs among gyne gut, ovaries and mesosoma tissues. (A) *Atta cephalotes,* (B) *Atta sexdens*, (C) *Acromyrmex echinatior* and (D) *Mycetomoellerius mikromelanos*. Each row represents a single OTU (*Wolbachia* OTU1, *Mesoplasma* OTU2, *Spiroplasma* OTU3, *Amycolatopsis* OTU5 and *Pseudonocardia* OTU10) and each column represents an individual gyne ant separated into the colonies they were taken from. Colony names are simplified as numbers; see S1 Table for colony collection codes. Samples from young queens are excluded here. The color scale is based on log base 4 transformation of relative (proportional) abundance out of 5000 reads.

Known bacterial endosymbionts were consistently found in/on gyne tissues and were represented by the three most abundant OTUs in our dataset, belonging to the genera *Wolbachia*, *Mesoplasma* and *Spiroplasma* (Fig 4). While we recovered a number of rare additional OTUs from the same bacterial genera, a single OTU was always abundant for each symbiont genus. The most abundant OTU across all samples, *Wolbachia*, was part of the core community in all gyne-associated tissues of *A. sexdens* and *Ac. echinatior*, and was present at ~75% relative abundance in most *Ac. echinatior* gyne tissue samples (Fig 4 and S4 Table). The second most abundant OTU across all samples was identified as *Mesoplasma* which shares 99% sequence similarity with the 16S rRNA gene of a previously identified symbiont of fungus-growing ants, EntAcro1 (GenBank accession KR336618) [38]. *Mesoplasma* constituted a large proportion of the bacterial communities associated with *A. cephalotes* and *Ac. echinatior* gyne guts and ovaries (Fig 4). This OTU was a core taxon in gyne guts for all three leafcutter species (S4 Table) but was less abundant in *A. sexdens*. Although the same *Mesoplasma* OTU was sometimes identified in *M. mikromelanos*, *Spiroplasma* (OTU3) was more consistently present and a member of the core community in gyne guts and ovaries of this species (Fig 4D). This OTU shared ~98% sequence similarity with the *Spiroplasma* in the guts of *M. mikromelanos* (previously *zeteki*) and *S. amabilis* workers [42]. Rhizobiales (OTU7 and OTU8) were also found in queen tissues but were not a part of the core community in any species (S4 Table). Actinomycetota were present in gyne mesosoma samples of all ant species but were most abundant in *M. mikromelanos* and *Ac. echinatior* (Fig 4 and S3 Fig). The primary genus of Actinomycetota associated with fungus-growing ants, *Pseudonocardia* (OTU10), was a core member of the *Ac. echinatior* queen mesosoma microbiome (S4 Table) and was highly abundant in gynes of that species (Fig 4C). A close relative to *Pseudonocardia* and another symbiont of fungus-growing ants, *Amycolatopsis* (OTU5), was a core member of all queen and gyne tissues in *M. mikromelanos* (S4 Table) and was consistently abundant in the mesosoma samples of gynes in this species (Fig 4D). Actinomycetota made up a smaller proportion of the overall mesosoma community in *Atta* queens/gynes and were not among the core taxa in those species (S3 Fig and S4 Table). Although not consistently present in either *Atta* species, the same two Actinomycetota OTUs mentioned above were components of gyne bacterial microbiota in at least one sample from every *Atta* colony sampled and were sometimes relatively abundant in *A. sexdens* (Fig 4B). The *Pseudonocardia* OTU (OTU10) was > 97% similar in sequence to *Pseudonocardia* previously isolated from multiple fungus-growing ant species [46–48]. Overall, our results support our H2 hypothesis that ant-associated bacterial symbionts can be vertically transmitted by gynes/founding queens.

### Pellet and queen-associated bacterial communities have host-ant-specific signatures

While hypotheses H1 and H2 address vertical transmission of garden and ant-associated symbionts, respectively, H3 emphasizes possible interspecific similarities and differences in bacterial communities which have the potential to be vertically transmitted. Five OTUs were identified as core members of unmated gyne pellet communities in all ant species sampled: *Moraxella* (OTU14), *Staphylococcus* (OTU15), *Lawsonella* (OTU20), *Pelomonas* (OTU21), and *Micrococcus* (OTU27) (S4 Table). The representative sequences of these shared OTUs are most similar to sequences from bacteria which are human-associated or environmentally isolated (S2 Table).

**Fig 5:**
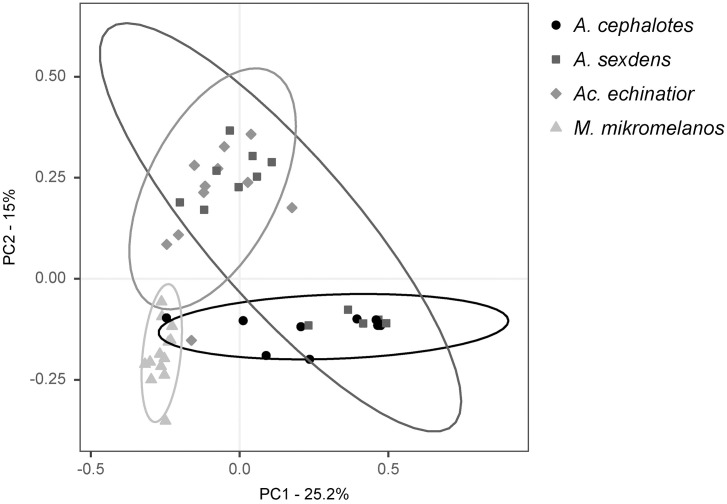
Between-species comparisons of pellet beta-diversity using Weighted Unifrac Principal Coordinate Analysis (PCoA). Shapes and shades of black/grey designate the ant species from which individual pellets were sampled (*Atta cephalotes*, *Atta sexdens*, *Acromyrmex echinatior*, and *Mycetomoellerius mikromelanos*) and ellipses represent 95% confidence intervals. *M. mikromelanos* pellets hosted different bacterial microbiota than those of the three leafcutter species (p = 0.006).

We found no significant pairwise differences in garden microbiota between species, aside from between *M. mikromelanos* and *Ac. echinatior* (p < 0.05) (S6 and S7 Tables). Across all four species, we found that the composition of pellet bacterial communities differed significantly (p < 0.01) (S6 and S7 Tables). Specifically, post-hoc tests show that *M. mikromelanos* pellet bacterial communities were significantly different from those of the three leafcutter species (p < 0.01), and that pellets of *Atta* species were more similar to each other than any pairwise comparison between different genera (p > 0.05) (Fig 5, S6 and S7 Tables). However, pellets from two colonies of *A. sexdens* shared similar bacterial microbiota with *Ac. echinatior*, so the *A. sexdens* samples appeared as two distinct clusters in the PCoA ordination plot, though this separation is less pronounced when visualized with nMDS (Fig 5 and S5 Fig). Pellet bacterial communities were not significantly different between colonies within each ant species, apart from the above-mentioned two colonies of *A. sexdens* (p < 0.05) (S6 and S7 Tables).

**Fig 6:**
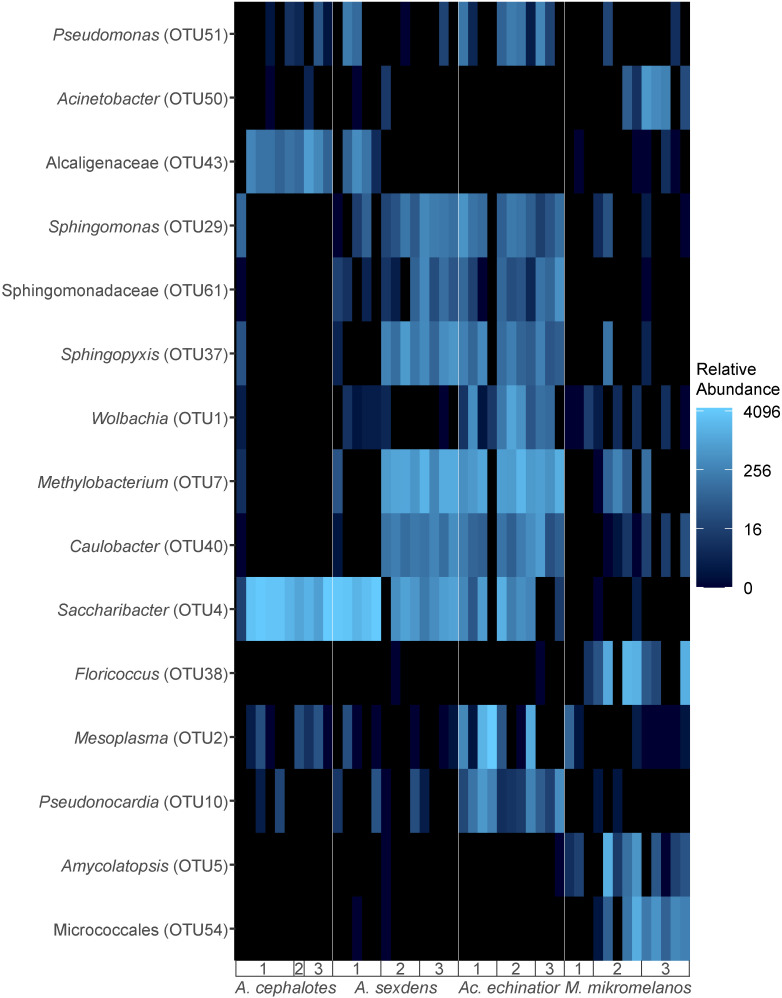
Heatmap depicting variation in abundance of bacterial OTUs both driving interspecific differences in pellet microbiota and associated with particular species. Only the OTUs which contributed to statistically significant differences in beta-diversity between ant species are shown. These OTUs contribute the most to differences in beta-diversity between ant species through SIMPER analysis and are also significantly associated with particular ant species through indicator species analysis. The OTUs on the Y-axis are ordered by clustering with Principal Coordinate Analysis (PCoA) using weighted Unifrac distances. The heatmap color scale is based on log base 4 transformation of relative (proportional) abundance out of 5000 reads. See S1 Table for corresponding colony collection codes.

SIMPER analysis showed that the differences in beta-diversity between *M. mikromelanos* pellet bacterial communities and those of the other three ant species were driven by a few abundant bacterial taxa, such as *Saccharibacter* (OTU4), *Amycolatopsis* (OTU5), *Floricoccus* (OTU38) and an unidentified Micrococcales (OTU54) (Fig 6 and S8 Table). Particularly OTU4 (*Saccharibacter*) contributed significantly to differences in pellet beta-diversity across ant genera and accounted for ~3.5-25% of dissimilarity between these communities (S8 Table). *Saccharibacter* is an indicator species for both *Atta* species (S9 Table*). Saccharibacter* is also a member of the core pellet community for all three leafcutter species (S4 Table) and was particularly abundant in gyne pellets of some *Atta* colonies (Fig 6). OTU4 is most closely related to uncultured bacteria isolated previously from leafcutter ant bodies and refuse dumps (99.2% similarity to GenBank accessions KF248837.1 and LN568338.1) but relatively distantly related to any other GenBank reference sequences (~91% sequence identity to *Saccharibacter floricola*) (S2 Table).

**Fig 7:**
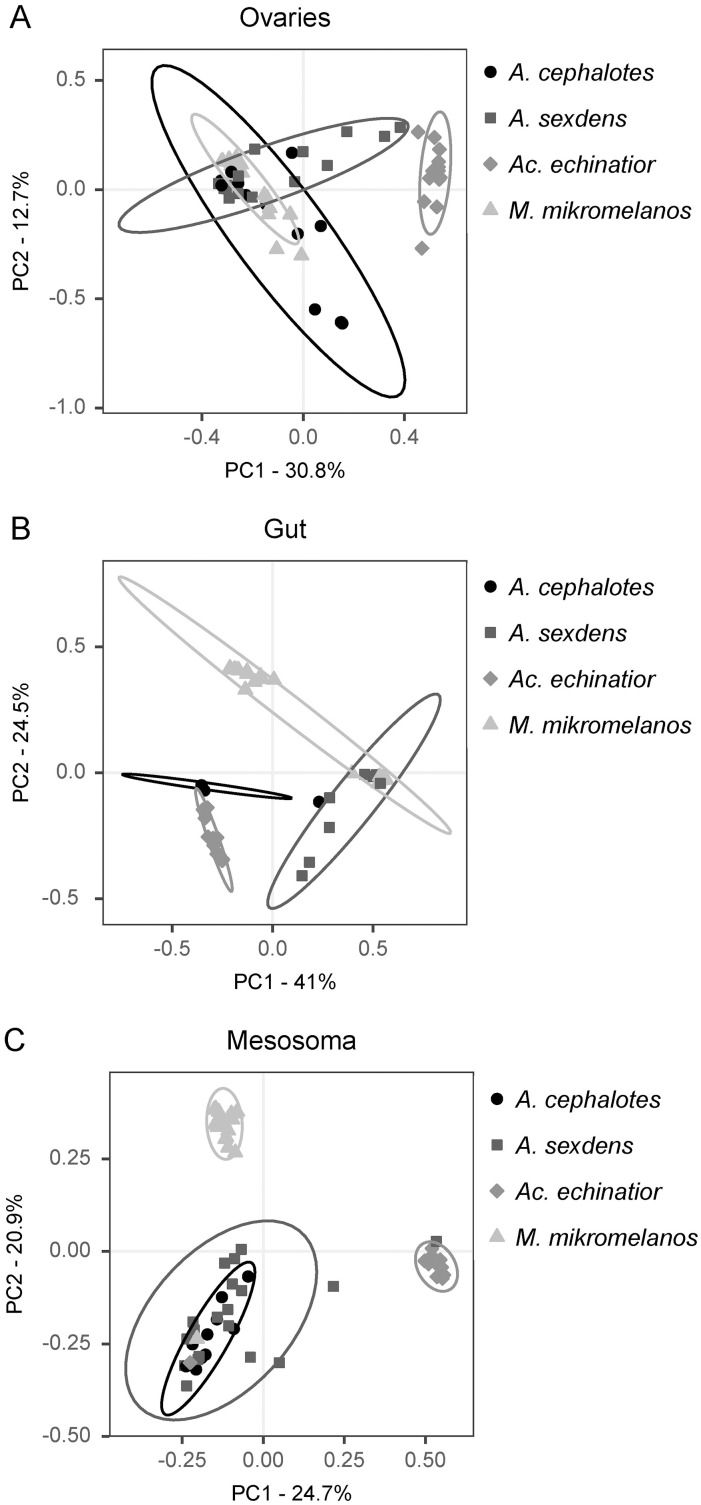
Between-species comparisons of beta-diversity across queen/gyne tissues using Weighted Unifrac Principal Coordinate Analysis (PCoA). (A) Gynes/queens of all species hosted distinct bacterial communities in their ovaries (p < 0.05). (B) Gynes/queens of all species also hosted distinct bacterial communities in their guts (p < 0.05). Four *A. cephalotes* gyne gut samples from colony Ac2 are directly overlaid in the plot as these samples were dominated by *Mesoplasma*. (C) The mesosomas of the two species of *Atta* were indistinguishable (p = 1). *Ac. echinatior* gynes and *M. mikromelanos* gynes/queens hosted distinctly different bacterial communities on their mesosomas (p = 0.006). Samples of unmated gynes and queens from *A. sexdens* and *M. mikromelanos* are analyzed together. Ellipses represent 95% confidence intervals.

We also found host-specific signatures in microbial communities associated with queen and gyne ovaries, guts and mesosomas (Fig 7). Ovaries hosted distinctly different microbiota across all four ant species (p < 0.001) (Fig 7A, S6 and S7 Tables). Further, gut microbiota were also distinct between all four ant species (p < 0.05) (Fig 7B and S6 Table). While *Wolbachia* and *Mesoplasma* were shared across ant species, SIMPER analysis indicates that the three most abundant OTUs contribute the most to these differences in both gut and ovary microbiota composition between species (S8 Table). *Wolbachia* was found to be both an indicator species for *Ac. echinatior* guts, ovaries, and mesosomas as well as a major driver of differences in beta-diversity between the queen tissues of *Ac. echinatior* and the other ant species in this study (S8 and S9 Tables). *Spiroplasma* is an indicator species of *M. mikromelanos* guts and ovaries, while *Mesoplasma* is an indicator species for both *Ac. echinatior* and *A. cephalotes* guts and ovaries (S9 Table). The beta-diversity analysis for queen and gyne mesosoma microbiota showed that mesosoma microbiota varies by ant genus, visualized by distinct clusters for *M. mikromelanos* and *Ac. echinatior* and a lack of differentiation between the mesosoma microbiota composition of two *Atta* species who do not consistently host Actinomycetota (Fig 7C, S6 and S7 Tables). Along with the three most abundant OTUs, the primary Actinomycetota OTUs *Amycolatopsis* (OTU5) and *Pseudonocardia* (OTU10) contributed to much of the interspecific differences in mesosoma microbiota composition (S8 Table). Specifically, *Amycolatopsis* (OTU5) was an indicator species of *M. mikromelanos* while *Pseudonocardia (*OTU10) was an indicator species of *Ac. echinatior* (S9 Table). Rhizobiales (OTU7 and OTU8) did not show as strong of ant host-specificity as the above-mentioned bacterial groups, although Rhizobiaceae (OTU8) contributed to differences in queen gut and mesosoma microbiota between *Ac. echinatior* and other ant species (S8 Table). Overall, we found that fungus-growing ant queen and gyne ovaries, guts and mesosomas hosted species and/or genus-specific bacterial communities even though some bacterial taxa were shared across species.

## Discussion

In this study we tested three hypotheses to develop a better understanding of the transmission dynamics of fungus-growing ant bacterial microbiota. We first hypothesized that garden-associated bacteria are vertically transmitted via the fungal cultivar inoculum (i.e., pellet) which fungus-growing ant queens use to start a new fungal garden. Despite gardens and queen pellets hosting distinct microbiota, we found a few bacterial OTUs that were core taxa in both pellets and gardens within our four study species. For example, *Klebsiella*—a nitrogen fixer in *Atta* and *Acromyrmex* ant gardens [23]—appears to be transmitted via queen pellets in *Ac. echinatior* and *M. mikromelanos*. Thus, our results indicate that only a select few garden-associated bacteria are consistently transmitted by queens, offering some support for our first hypothesis (H1). Further, previously documented fungus-growing ant-associated symbionts, *Wolbachia*, *Mesoplasma* and *Spiroplasma* [30,34,38], were consistently found in pellet and garden samples, as was Rhizobiales in *A. cephalotes* [39,40]. This suggests that the fungus garden may serve as a reservoir facilitating vertical transmission via queen pellets for both ant- and garden-associated symbionts [24,49], indicating a close relationship between the ants and these bacterial associates. Additionally, since the fungus-growing ant queens deposit fecal droplets when feeding and tending their garden, both queen gut bacteria and pellet inocula likely contribute to the initial assembly of bacterial communities in the gardens of incipient ant nests [34,50,51]. Future work should elucidate the functional role, if any, of these bacteria found in fungus gardens.

Overall, our results support our second hypothesis (H2) that known mutualists of attine ant workers are vertically transmitted by queens to new offspring. We found that only one OTU belonging to each of the previously identified endosymbiont genera (*Wolbachia*, *Mesoplasma* and/or *Spiroplasma*) was present at a high abundance in our dataset. Other bacteria previously found to be facultatively associated with fungus-growing ant guts, such as Rhizobiales, were found in the tissues of queens but not consistently (i.e., present in at least 65% of samples). Our results corroborate previous work suggesting complete transovarial transmission of *Wolbachia* [41]. However, contrary to a recent study, which suggested that *Atta sexdens* from Brazil do not consistently associate with *Mesoplasma*, we found these bacteria to be a core taxon in the guts of *A. sexdens* and *A. cephalotes* queens [52]. While Mycoplasmatales symbionts may not be transmitted consistently via eggs, their abundance and prevalence in gardens, worker guts, and gyne guts remains highly relevant. We hypothesize that trophallaxis between adults and reproductive larvae mediates the transmission of Mycoplasmatales symbionts, as shown for transmission of bacteria in other social arthropods [8,53,54].

We also found support for vertical transmission of Actinomycetota by *Acromyrmex* and *Mycetomoellerius* queens, as species-specific Actinomycetota genera were consistently present on queen mesosomas. Previous work also presents evidence of vertical transmission of Actinomycetota in fungus-growing ants, such as visible biofilms on young queen exoskeletons (personal observation) and the presence of co-evolved crypts on both worker and queen exoskeletons which maintain Actinomycetota biofilms [55–57]. Our results indicate some level of host specialization, as *Pseudonocardia* is mostly associated with *Acromyrmex* while *Amycolatopsis* is mostly associated with *Mycetomoellerius*. While there is evidence of fungus-growing ant species-specificity in Actinomycetota associations, repeated horizontal transmission events have also occurred [27,28,46]. The same Pseudonocardiaceae (Actinomycetota: Actinomycetes: Pseudonocardiales) OTUs were found across all four ant species, despite the lack of structures that support known Actinomycetota biofilms on *Atta* cuticles [55,58,59]. Interestingly, recent evidence suggests that cuticular microbiota in *Atta* do not play a defensive role, at least against the *Metarhizium anisopliae* fungal entomopathogen [60]. While multiple Actinomycetota strains can co-exist on fungus-growing ant worker cuticles, we found that only one or two OTUs dominated the bacterial communities of queen and gyne mesosomas, similar to what has been previously shown on cuticles of callow workers of *Acromyrmex* leafcutter ants [27]. It is important to note that *Pseudonocardia* has also been found in the guts of sympatric Panamanian *M. mikromelanos* [30,45], so results obtained from whole (parts of) body extracts should be interpreted accordingly.

Despite ample opportunities for fungal gardens (and thus infrabuccal pellets) to acquire bacterial associates horizontally from the environment, we identified a set of core pellet bacterial OTUs with as of yet unknown functions that were shared across the four Panamanian fungus-growing ant species (*Moraxella*, *Staphylococcus*, *Lawsonella*, *Pelomonas*, and *Micrococcus*). While none of these five genera have been previously identified as fungus-growing ant symbionts, bacteria in the genus *Staphylococcus* have been found in earlier fungal garden studies [24,61,62] and may function to increase bioavailable iron and promote growth of the fungal cultivar [62]. *Pelomonas* (OTU 21) is closely related to bacterial OTUs from a variety of sources, but particularly relevant in the context of our study is the association of this bacterial genus with fungal gardens of *Mycocepurus goeldii* [63] and the males of *Trachymyrmex septentrionalis* [64]. The taxa in infrabuccal pellets of attine dispersing queens suggests they could be facultative symbionts, but this remains to be tested. We believe the presence of these bacteria is unlikely to be a result of contamination as they are not found in our sequencing blanks nor consistently in other sample types. Further, this shared set of core bacterial taxa in pellets across attines suggests that there may be unknown mechanisms conserved across attines that influence which garden bacteria are vertically transmitted. Overall, our results corroborate previous evidence that bacterial associates of fungal gardens are acquired through both vertical and horizontal transmission [65].

Although we identified a shared set of core bacteria across the infrabuccal pellets of all four species investigated, our results suggest ant species-specificity in pellet microbiota. We found that the bacterial microbiota of *M. mikromelanos* pellets were distinct from those of the three leafcutter ants and pellet microbiome composition of the two *Atta* species sampled were not distinct from each other, supporting our third hypothesis (H3). Known fungus-growing ant symbionts (e.g., *Wolbachia* and Actinomycetota) are more associated with the pellets of some species than others, indicating an effect of the host ant species on the likelihood that certain bacterial symbionts will be transmitted to the next generation. This specificity in pellet microbiota mirrors the divergence in attine ant abdominal microbiomes that arose when the ancestors of the *Paratrachymyrmex* sister genus of *Mycetomoellerius* colonized Central and North America [30,66]. The significant ecological differences between *M. mikromelanos* and sympatric leafcutter ants may also have influenced the vertical transmission dynamics of their microbiota. For instance, fungal cultivar clade, ant-associated microbiomes, and preferred forage material are different between *M. mikromelanos* and the three leafcutter ant species in this study [10,30,67]. They cultivate fungi from two sister clades, with the three leafcutter species cultivating a single species, i.e., *Leucoagaricus gongylophorus*, and *M. mikromelanos* cultivating a related but undescribed species of fungus [11,67,68]. Thus, the differences in pellet microbiota may thus be a consequence of the selective environment imposed by the fungal cultivar strain. Further, pellet microbiome composition may also be influenced by the bacteria associated with the plants the ants are gathering to feed their cultivar, as small fragments of plant material were visible in the pellets of the leafcutter species sampled (personal observation). While we did not find a strong ant host signature in the garden microbiota, our garden sample size was relatively small, so future work should include more within-nest samples considering the potential for undetected variation in bacterial communities [61].

### Conclusions

Overall, our results provide evidence that the role of attine ant queen infrabuccal pellets is more complex than just vectoring an inoculate of a queen’s maternal fungal cultivar to the next generation. Recent studies have characterized the bacterial microbiomes of the digestive systems of attine ant workers [30,34,41] and fungal gardens of mature colonies [13,21,65,69,70]. These have clarified that several bacterial genera have specific functions in the farming symbiosis, suggesting a close ecological relationship with their host ants [23,38–40]. These persistent associations suggest vertical transmission of bacteria by queens, but *de novo* acquisition of bacteria due to foraging later in colony life cannot be ruled out. Our study is the first to target this question by direct sampling of the eggs and infrabuccal fungus garden pellets of multiple species of attine ants. While we sampled only four sympatric attine ant species from a single Panamanian location and our sample sizes were modest, our results demonstrate that bacteria with previously proposed complementary functions in this complex farming symbiosis have the potential to be vertically transmitted via queens through multiple routes. Although, horizontal transmission of bacteria is also likely, especially in fungal gardens where active foraging is taking place.

We also identified several bacterial lineages with unknown functions that are consistently vertically transmitted and where further work might elucidate a functional role. We hope that future work can build from our comparative approach and test the generality of our results across a larger assembly of fungus-growing ants with a wider geographic distribution. Methods developed for studying fungus-growing ant gut bacteria provide directions for additional studies to further test our hypotheses. For example, fluorescent in situ hybridization (FISH) microscopy using OTU or strain specific probes can be used to visualize and quantify bacterial abundance that can further be validated with quantitative PCR [30]. Experimental inoculation with genetically modified bacterial strains that express visible markers (e.g., fluorescent proteins) could be used to confirm the inferred pellet-mediated vertical transmission of these bacteria to new gardens. Strain-level assays, such as culturomics using generic and species-specific media, coupled with single-cell genomics could also be used to unravel the hidden diversity of individual bacterial strains that are transmitted by founding queens from natal gardens. Overall, our work adds support for vertical transmission of fungus-growing ant microbiota and contributes to a growing body of research elucidating the relationships between these ants and their bacterial associates.

### Materials and methods

#### Sample collection.

*Atta sexdens* (n = 3 colonies, 14 gynes) *A. cephalotes* (n = 3 colonies, 13 gynes), *Ac. echinatior* (n = 3 colonies, 13 gynes), and *M. mikromelanos* (n = 3 colonies, 14 gynes) samples were collected from field nests in the Panama Canal region in May 2014 (S1 Table). Gynes (unmated female reproductives destined to become queens) were collected from live colonies immediately or < 7 days after they were excavated from the field. Infrabuccal pellets were dissected from their oral cavity using sterilized forceps and a stereomicroscope, then preserved dry at −80°C until DNA extraction. Nest-founding queens were collected in the same time period for *A. sexdens* (n = 5 colonies) and *M. mikromelanos* (n = 6 colonies) (S1 Table). In these cases, eggs and incipient fungal garden material were also sampled. In order to preserve queen-derived microbes coating the eggs, they were not surface sterilized before DNA extraction. These small gardens presumably originated from the pellet inocula that queens disperse with. Garden samples were collected from a queen-dug cavity and were fed and nurtured by the young queen. Some may have had plant material recently incorporated into the garden substrate. Fungal garden samples were collected directly upon field sampling and stored at −80°C until DNA extraction.

Single guts (midgut and hindgut), and reproductive tracts for queens, were dissected from the gaster in sterile phosphate-buffered saline (PBS) under a stereomicroscope. Although our target in dissecting queen reproductive tracts was the ovaries, connected reproductive tract organs (i.e., spermathecae) were not removed to preserve sample integrity. The rest of the body (i.e., mesosoma) was stored separately so that it was possible to consider the different sources of queen microbiomes. Worker ants (n = 1–2/colony) were also sampled from each of the mature colonies from which gynes were sampled in order to dissect gut and mesosoma tissue for comparison.

#### Molecular methods.

DNA was extracted from frozen queen guts, ovaries, queen mesosomas, and eggs (n = 38, 64, 64, and 8 respectively) using the DNeasy blood and tissue kit (Qiagen) following the manufacturer instructions. DNA from frozen fungal garden and pellet samples (n = 27 and 51 respectively) was extracted with a Cetyltrimethylammonium Bromide (CTAB) solution followed by a phenol–chloroform extraction [71]. We used MilliQ water as a negative control (n = 5) to assess for sample contamination during DNA extractions. We also included a mock community (BEI Resources, Manassas, VA, USA) extraction to assess error rates. PCR primers 515F (5’- GTGCCAGCMGCCGCGGTAA −3’ and 806R (5’- GGACTACHVHHHTWTCTAAT −3’) covering the V4 region of the 16S rRNA gene were used for PCR reactions using a mix containing 2.0µl 10 × AccuPrime™ PCR Buffer II (15 mM MgCl2), 0.15µl AccuPrime™ Taq DNA Polymerase (2 units/µl, Life Technologies), 1.0µl of each primer (10 µM), 1.0µl diluted template and autoclaved MilliQ water to a total of 20µl. The following thermal profile was used: 95°C for 2 min, followed by 30 cycles of 95°C for 20 s, 55°C for 15 s, 72°C for 5 min, and final extension at 72°C for 5 min. All samples were re-eluted in 50–150µl AE buffer (Qiagen) then quantified using an ND‐1000 spectrophotometer (NanoDrop Technologies, Wilmington, DE, USA). The purified amplicons were then pooled in equimolar concentrations. Extracted DNA was sent to Microbial Systems Laboratory at the University of Michigan (Ann Arbor, MI) for library preparation and 16S rRNA sequencing using the Illumina Miseq sequencer with the v3 kit (2x300cycles) as described in [72].

#### Sequencing data processing.

Sequences were processed with mothur v1.44.3 [73]. The raw sequences were filtered using a minimum quality score of 25 and reads were discarded when longer than 275 bp in length or showing any ambiguous bases. Unique sequences were then aligned to a non-redundant database (SILVA v138) customized for the 16S rRNA gene V4 region and any sequences which did not cover the V4 hypervariable region were removed. Unique sequences with a homopolymer length above eight were subsequently removed. Unique sequences were screened again after removing overhangs. Chimeric sequences were identified using vsearch v. 2.13.3 and removed. Filtered sequences were identified taxonomically using the same customized SILVA database (with an 80% bootstrap confidence threshold) and any sequences classified as Archaea, Eukaryota, mitochondria, chloroplast or unknown were excluded. Bacterial sequences were clustered de novo into operational taxonomic units (OTUs) using the average neighbor algorithm with a 97% similarity threshold while retaining singletons. The final sequence file consisted of 9,271 filtered and unique OTUs. The taxonomy of the 50 most abundant OTUs was verified manually using NCBI BLASTn (S2 Table). Analysis of the sequences from our blank control samples did not indicate significant contamination, so these samples were removed from the dataset.

#### Data analyses.

Rarefaction and diversity analyses were completed in R v. 3.6.2 with the packages phyloseq v1.38 and vegan v2.5.7 using the OTU table, taxonomy and tree files imported from mothur [74,75]. The OTU abundance and taxonomy data can be found in the S3 Table. Rarefaction curves were visualized using vegan and ranacapa v1.0 R packages [74,76]. All samples were normalized by randomly subsampling to 5,000 reads, reducing the sample number to 268 (28 samples had < 5000 reads) and reducing the total number of OTUs from 9,271–6,306. This dataset was used for all downstream analyses. While 13–14 gynes and 5–6 nest-founding queens (for 2 of 4 species sampled) were sampled for each ant species, for some individual ants, sample(s) from different tissue types were not included in the bulk of analyses due to having less reads than the cutoff used to rarefy the data. For this reason, we also excluded seven garden samples and four pellet samples. Alpha-diversity, or within sample diversity, was evaluated using the Inverse Simpson index since the Shannon index is affected by low sequencing coverage. Differences in alpha-diversity between groups were determined by non-parametric Kruskal-Wallis and post hoc Wilcoxon rank sum tests. Following the methods of Meirelles et al. 2016 [24], we considered a bacterial OTU as part of the core microbiota if this taxon was recovered from at least 65% of the samples, analyzing each of the tissue-types and ant species separately using the microbiome v1.16 R package [77]. We constructed heatmaps to visualize variation in relative abundance of known fungus-growing ant symbiont OTUs in queen tissues as well as OTUs which may be transmitted by queens (through infrabuccal pellets or by transovarial means) using the packages phyloseq and NeatMap v0.3.6.2 [78]. We also constructed bar plots using ggplot to visualize phylum-level bacterial microbiota composition for each tissue sampled in each ant colony.

Beta-diversity, or dissimilarity between samples, was assessed using Bray-Curtis dissimilarity and weighted Unifrac distances and evaluated using analysis of similarity (ANOSIM) and permutational multivariate analysis of variance (PERMANOVA, permutation number = 999) in vegan. Pairwise comparisons were performed with Bray-Curtis dissimilarity and weighted Unifrac distances and subjected to Bonferroni adjustment (package RVAideMemoire v0.9.81, function pairwise.perm.manova) [79]. Worker mesosoma and gut communities were not significantly different from those of queens or unmated gynes and were excluded from further beta-diversity comparisons to focus on vertically transmitted bacteria. Differences in weighted Unifrac beta-diversity distance values were visualized using the Principal Coordinate Analysis (PCoA) and nMDS (non-metric multidimensional scaling) ordination methods using the R package vegan. Similarity percentage analysis (SIMPER) based on Bray-Curtis dissimilarity distances implemented in the vegan package followed by non-parametric Kruskal-Wallis tests with false discovery rate (FDR) corrected p-values were used to identify OTUs responsible for statistically significant differences in bacterial communities across ant species, tissues, and colonies within species (function: simper.pretty v1.1 in R doi.org/10.5281/zenodo.4270481) [80]. We then constructed a heatmap using methods described above to visualize relative abundances of the OTUs which significantly contributed to differences in beta-diversity of pellet bacterial microbiomes across ant species. For ecologically relevant clustering in this heatmap, the y-axis was ordered with Principal Coordinate Analysis (PCoA) using weighted Unifrac distances rather than by hierarchical cluster analysis which has less ecological meaning [78]. Specific OTUs more likely to be associated with certain ant species were identified by indicator species analysis using the R package ‘indicspecies’ [81]. We also constructed a heatmap to visualize relative abundance of five abundant OTUs which belong to known fungus-growing ant symbiont genera in queen ovary, gut and mesosoma tissues.

#### Ethics statement.

Collection and export permits for samples used in this study were obtained from Panama’s National Authority for the Environment (Autoridad Nacional del Ambiente) (SE/A-41–13 and SEX/A-28–13, respectively).

### Supporting information

S1 Fig Rarefaction analyses of observed species richness as a function of sequencing depth for each tissue examined. Color indicates ant species. All samples in this study are represented, including those omitted from downstream analysis (e.g., workers and samples with <5000 reads).
(TIF)

S2 Fig Comparison of alpha-diversity using the Inverse Simpson index of bacterial communities associated with queens across sample types within four ant species. Bars above plots indicate significance values for non-parametric Kruskal-Wallis and post hoc Wilcoxon rank sum tests for differences between species within each tissue type (* p ≤ 0.05, ** p ≤ 0.01). Note y-axis scale differs across plots. Sample sizes: Eggs (Mm: n = 5, As: n = 3), Ovaries (Mm: n = 18, Ae: n = 13, Ac: n = 13, As: n = 16), Garden (Mm: n = 10, Ae: n = 4, Ac: n = 2, As: n = 4), Pellet (Mm: n = 13, Ae: n = 11, Ac: n = 10, As: n = 13), Gut (Mm: n = 20, Ae: n = 13, Ac: n = 6, As: n = 9), Mesosoma (Mm: n = 20, Ae: n = 12, Ac: n = 10, As: n = 19).
(TIF)

S3 Fig Bacterial microbiomes of gyne and queen tissues in four fungus-farming ant species characterized by phyla. Samples are pooled by colony and species identifiers (As, Ac, Ae and Mm) on colony IDs are omitted for simplicity. Samples derived from young queens rather than unmated gynes are noted with an asterisk (*). Phyla which comprised <1% of a community in each sample were binned into a separate category.
(TIF)

S4 Fig Within-species comparisons of egg and ovary beta-diversity using Weighted Unifrac Principal Coordinate Analysis (PCoA). (A) *Atta sexdens* and (B) *Mycetomoellerius mikromelanos*. Ellipses represent 95% confidence intervals. The sample size for *A. sexdens* egg samples was too low to calculate a statistical ellipse, but eggs and ovaries microbiota were different in both ant species (p < 0.05).
(TIF)

S5 Fig Between-species comparisons of pellet beta-diversity using Weighted Unifrac non-metric multidimentional scaling (nMDS). Shapes and shades of black/grey designate the ant species from which individual pellets were sampled (*Atta cephalotes*, Atta sexdens, *Acromyrmex echinatior*, and *Mycetomoellerius mikromelanos*) and ellipses represent 95% confidence intervals. *M. mikromelanos* pellets hosted different bacterial microbiota than those of the three leafcutter species (p = 0.006).
(TIF)

S1 Table Metadata for each sample (n = 302). Samples with the total reads <5,000 are highlighted in yellow and were excluded from beta-diversity analyses.
(XLSX)

S2 Table Closest NCBI RefSeq relatives to sequences from top 50 OTUs. The top 50 most abundant OTUs were chosen from the rarefied dataset.
(XLSX)

S3 Table Taxonomic assignment and absolute abundance of bacterial OTUs observed. Samples highlighted in yellow are blank controls.
(XLSX)

S4 Table OTUs (at 97% sequence similarity) inferred to belong to the core microbiome within sample types examined for four fungus-farming ant species. Core microbiota are defined as taxa present in at least 65% of samples within a group. Mesosoma and gut core analyses are restricted to queen samples.
(XLSX)

S5 Table Alpha diversity statistics using the Inverse Simpson measure. Statistically significant values (p < 0.05) are bolded.
(XLSX)

S6 Table Permutational multivariate analysis of variance (PERMANOVA) using weighted Unifrac distances comparing bacterial communities. A) Comparison of pellet microbiota between ant species and between colonies within species. B) Comparison of garden, eggs and queen tissues between ant species. C) Comparison of pellet/garden samples and egg/ovaries samples within ant species. Statistically significant values (p < 0.05) are bolded.
(XLSX)

S7 Table Permutational multivariate analysis of variance (PERMANOVA) using Bray-Curtis dissimilarity distances comparing bacterial communities. A) Comparison of pellet microbiota between ant species and between colonies within species. B) Comparison of garden, eggs and queen tissues between ant species. C) Comparison of pellet/garden samples and egg/ovaries samples within ant species. Statistically significant values (p < 0.05) are bolded.
(XLSX)

S8 Table SIMPER analysis indicating significant contributions of individual OTUs to observed differences in beta-diversity between ant species within all sample types. Each sample type was tested as an independent analysis: A) Pellets, B) Guts, C) Ovaries, D) Mesosomas. No OTU contributions were statistically significant for either garden or egg samples.
(XLSX)

S9 Table Indicator species analysis indicating individual OTUs significantly associated with a single ant species or a group of species within all sample types. Each sample type was tested as an independent analysis: A) Pellets, B) Guts, C) Ovaries, D) Mesosomas, E) Garden and F) Eggs.
(XLSX)
