# Supplementary figures and images for: Bacterial communities of fungus-growing ant queens are species-specific and suggest vertical transmission
Source: PLoS One. 2025 Oct 7;20(10):e0306011. doi: 10.1371/journal.pone.0306011 (PMC12503253; doi:10.1371/journal.pone.0306011)

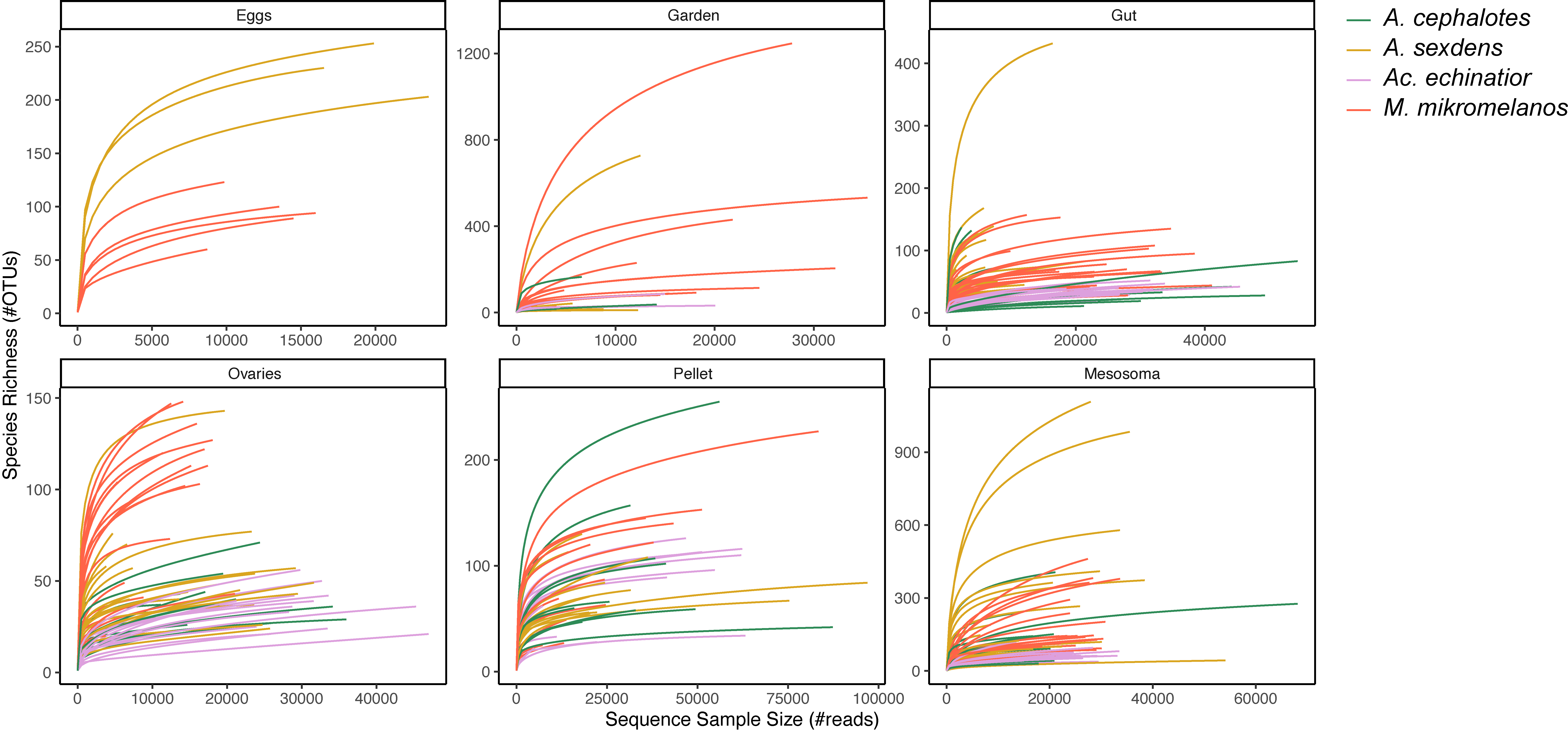

Supplement: S1 Fig — Color indicates ant species. All samples in this study are represented, including those omitted from downstream analysis (e.g., workers and samples with <5000 reads). (TIF) [file pone.0306011.s001.tif]

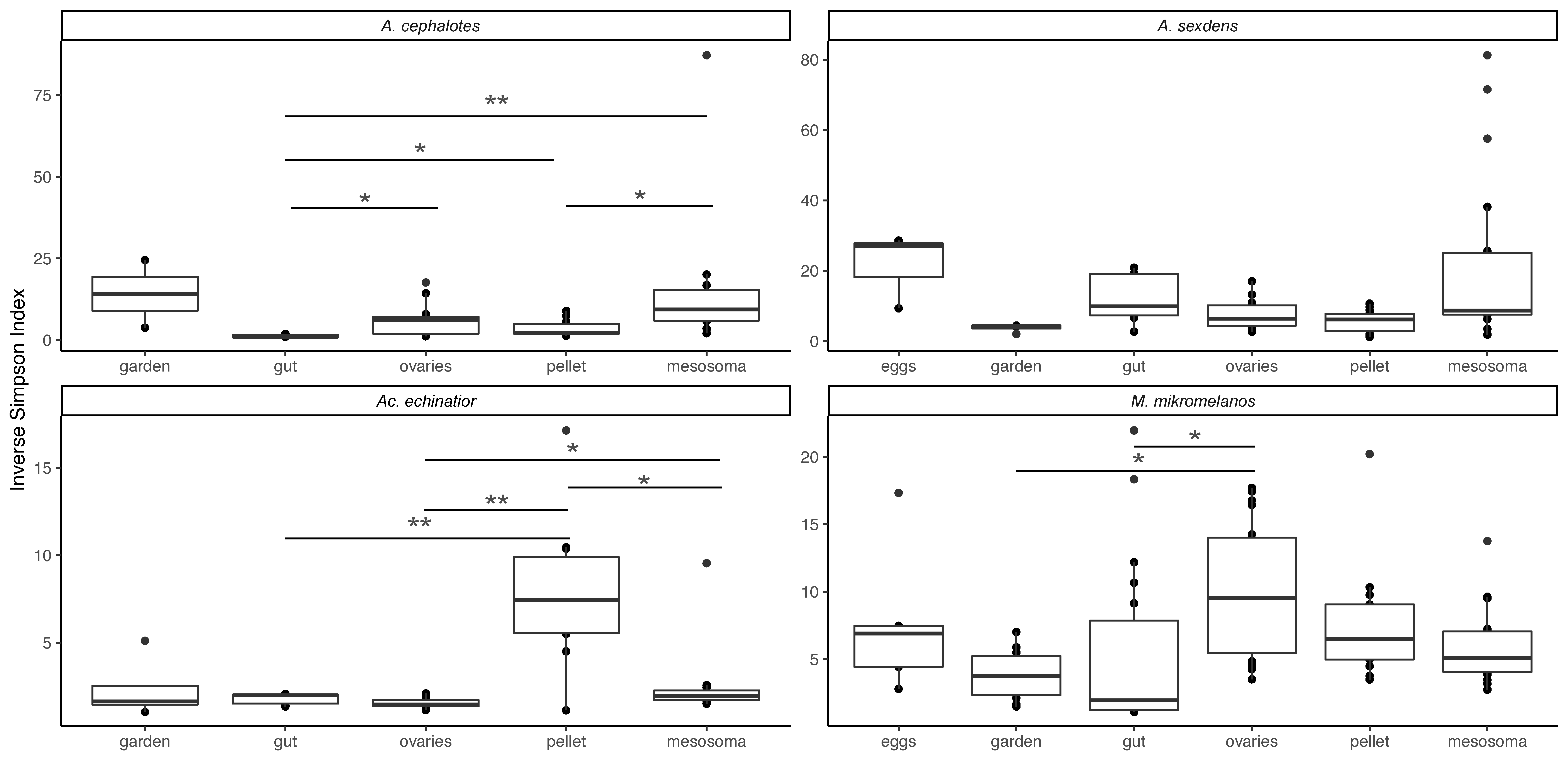

Supplement: S2 Fig — Bars above plots indicate significance values for non-parametric Kruskal-Wallis and post hoc Wilcoxon rank sum tests for differences between species within each tissue type (* p ≤ 0.05, ** p ≤ 0.01). Note y-axis scale differs across plots. Sample sizes: Eggs (Mm: n = 5, As: n = 3), Ovaries (Mm: n = 18, Ae: n = 13, Ac: n = 13, As: n = 16), Garden (Mm: n = 10, Ae: n = 4, Ac: n = 2, As: n = 4), Pellet (Mm: n = 13, Ae: n = 11, Ac: n = 10, As: n = 13), Gut (Mm: n = 20, Ae: n = 13, Ac: n = 6, As: n = 9), Mesosoma (Mm: n = 20, Ae: n = 12, Ac: n = 10, As: n = 19). (TIF) [file pone.0306011.s002.tif]

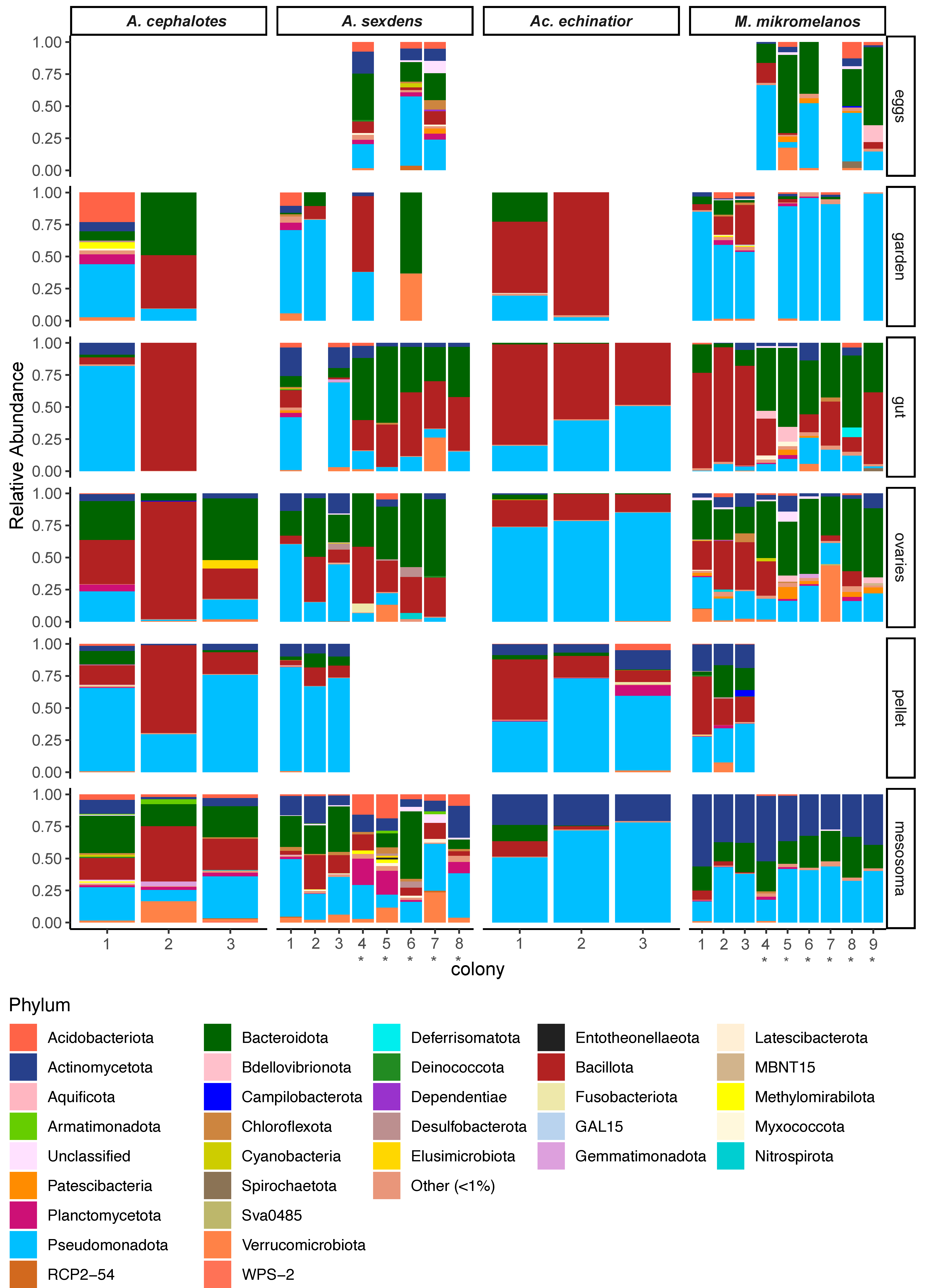

Supplement: S3 Fig — Samples are pooled by colony and species identifiers (As, Ac, Ae and Mm) on colony IDs are omitted for simplicity. Samples derived from young queens rather than unmated gynes are noted with an asterisk (*). Phyla which comprised <1% of a community in each sample were binned into a separate category. (TIF) [file pone.0306011.s003.tif]

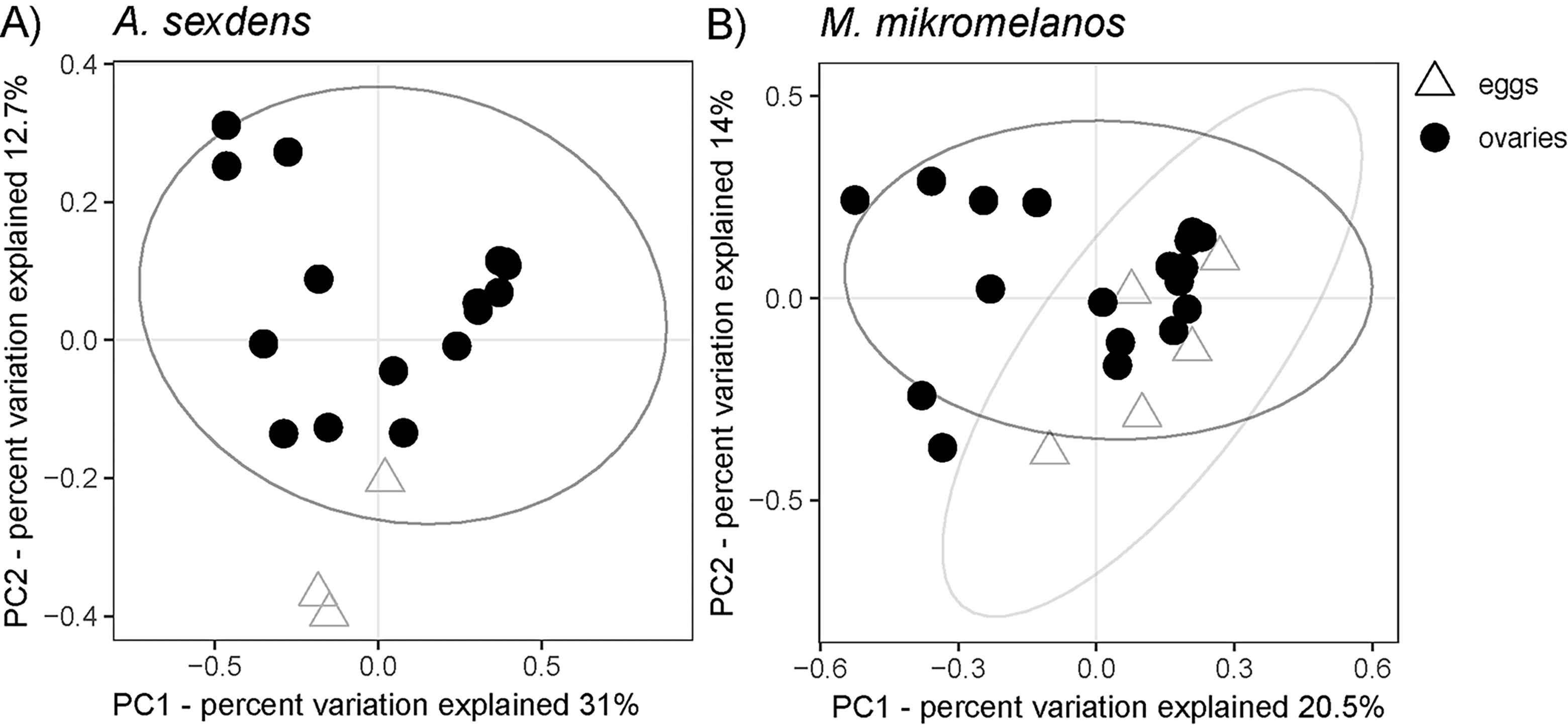

Supplement: S4 Fig — (A) Atta sexdens and (B) Mycetomoellerius mikromelanos. Ellipses represent 95% confidence intervals. The sample size for A. sexdens egg samples was too low to calculate a statistical ellipse, but eggs and ovaries microbiota were different in both ant species (p < 0.05). (TIF) [file pone.0306011.s004.tif]

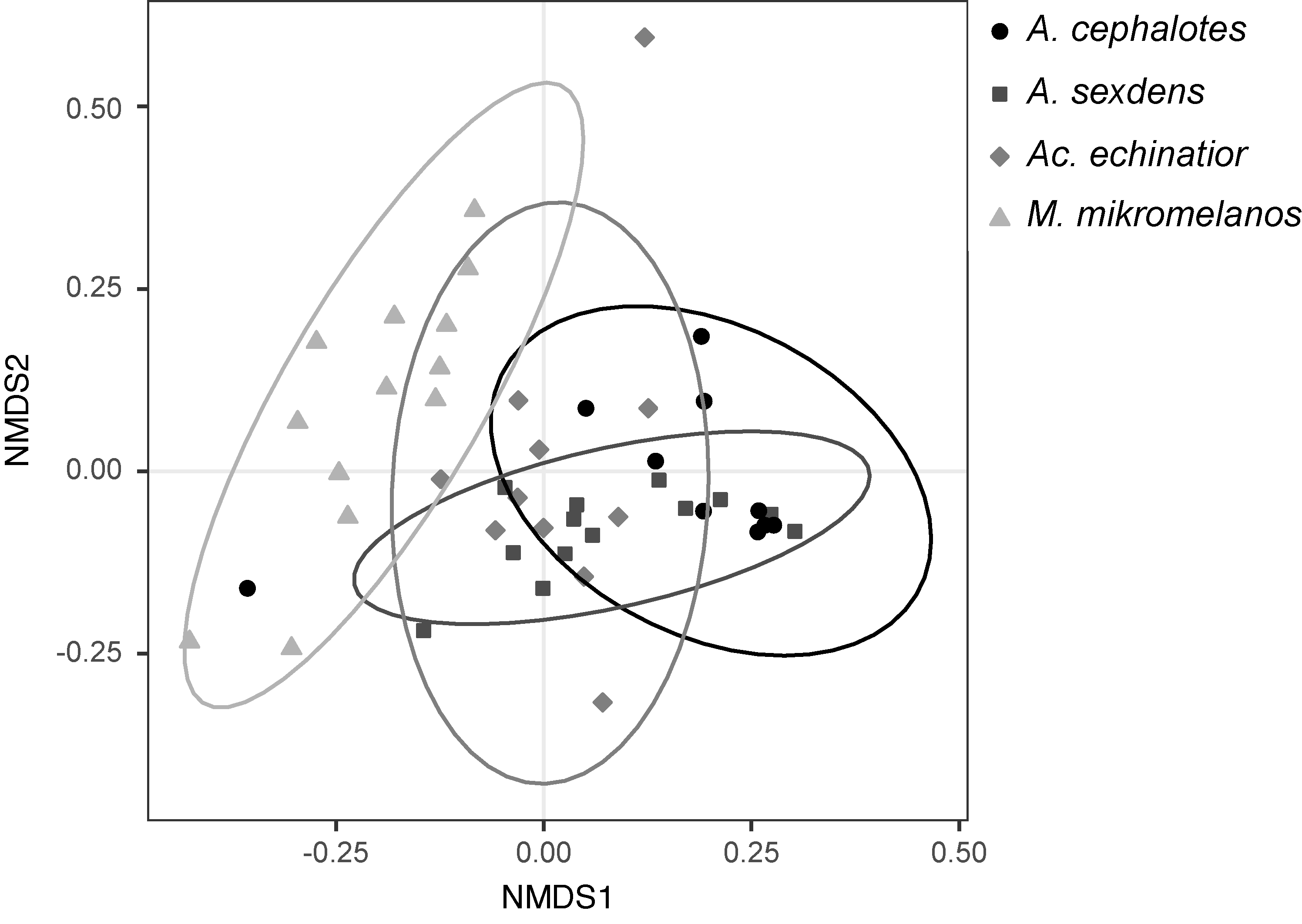

Supplement: S5 Fig — Shapes and shades of black/grey designate the ant species from which individual pellets were sampled (Atta cephalotes, Atta sexdens, Acromyrmex echinatior, and Mycetomoellerius mikromelanos) and ellipses represent 95% confidence intervals. M. mikromelanos pellets hosted different bacterial microbiota than those of the three leafcutter species (p = 0.006). (TIF) [file pone.0306011.s005.tif]
